# NLRP3 Inflammasome: A Potential Target in Isoflurane Pretreatment Alleviates Stroke-Induced Retinal Injury in Diabetes

**DOI:** 10.3389/fncel.2021.697449

**Published:** 2021-07-08

**Authors:** Hong-Bin Lin, Ying-Hui Lin, Jin-Yu Zhang, Wen-Jing Guo, Andrea Ovcjak, Zhi-Jian You, Zhong-Ping Feng, Hong-Shuo Sun, Feng-Xian Li, Hong-Fei Zhang

**Affiliations:** ^1^Department of Anesthesiology, Zhujiang Hospital of Southern Medical University, Guangzhou, China; ^2^State Key Laboratory of Ophthalmology, Zhongshan Ophthalmic Center, Sun Yat-Sen University, Guangzhou, China; ^3^Department of Physiology, Temerty Faculty of Medicine, University of Toronto, Toronto, ON, Canada; ^4^Department of Anesthesiology, Liuzhou People’s Hospital, The Affiliated Liuzhou People’s Hospital of Guangxi Medical University, Liuzhou, China; ^5^Department of Surgery, Temerty Faculty of Medicine, University of Toronto, Toronto, ON, Canada; ^6^Department of Pharmacology and Toxicology, Temerty Faculty of Medicine, University of Toronto, Toronto, ON, Canada; ^7^Leslie Dan Faculty of Pharmacy, University of Toronto, Toronto, ON, Canada

**Keywords:** ischemic stroke (IS), retinal injury, diabetes, NLRP3 inflammasome, isoflurane

## Abstract

Ischemic stroke remains a devastating disease which is the leading cause of death worldwide. Visual impairment after stroke is a common complication which may lead to vision loss, greatly impacting life quality of patients. While ischemic stroke is traditionally characterized by a blockage of blood flow to the brain, this may coincide with reduced blood flow to the eye, resulting in retinal ischemia and leading to visual impairment. Diabetes increases the risk of ischemic stroke and induces diabetic retinopathy; the latter may be more sensitive to the ischemic retinal injury. In diabetic status, the underlying mechanism in stroke-induced retinal injury has not been fully clarified. The NLR pyrin domain containing 3 (NLRP3) inflammasome is an important activator of inflammation, which may play a critical role in catalyzing and forming certain pro-inflammatory cytokines in both cerebral and retinal ischemia. Isoflurane has been demonstrated to inhibit the activation of the NLRP3 inflammasome and show neuroprotective effects. In this study, we established a diabetic mouse model and performed the middle cerebral artery occlusion procedure to induce ischemic stroke. Our results revealed that cerebral ischemia-induced retinal injury in the diabetic model. Isoflurane pretreatment alleviated the cerebral and retinal injury after ischemic stroke. Of note, isoflurane pretreatment inhibited the NLRP3 inflammasome activation in the retina, indicating that isoflurane pretreatment may provide substantial retinal protection in stroke-induced retinal injury in diabetes.

## Introduction

Stroke, the leading cause of death and disability, affects nearly 30 million people worldwide each year, with ischemic stroke accounting for upwards of 75% of all cases (Wang et al., 2017). Visual impairment is a common complication in stroke patients which affects functional recovery and quality of life (Sand et al., 2013). A previous study has reported that 92% of patients suffer from visual impairments after stroke, including eye movement disorders, visual acuity reduction, visual field impairment, and visual perceptual difficulty (Rowe, 2017). Visual impairments after stroke are also predictive of worse prognosis, stroke recurrence, and higher mortality (Deng et al., 2018; Sand et al., 2018). In rodent ischemic stroke models, visual impairments after stroke have also been widely reported which accompany neurological and retinal injury (Steele et al., 2008; Allen et al., 2014; Liu et al., 2015; Nguyen et al., 2019; Lee et al., 2020). The main pathogenesis of stroke-induced visual impairments includes higher visual center damage, retinal ischemia, and subsequent retinal damage (Steele et al., 2008; Rowe et al., 2013; Nguyen et al., 2019). Diabetes is a well-established independent risk factor related to cerebrovascular diseases and retinal ischemic injury (Ergul et al., 2014; Hendrick et al., 2015), which increases the incidence of ischemic stroke and visual impairments after stroke (Liu et al., 2016; Bragg et al., 2017; Sand et al., 2018). However, the underlying molecular mechanisms of stroke-induced retinal injury are still far from precisely dissected, especially in diabetic disease.

Inflammation plays an important role in the progression of a diabetic stroke, retinal ischemia, and diabetic retinopathy (Rivera et al., 2017; Hong et al., 2018; Forrester et al., 2020). The retina is an extension of the central nervous system and the pathological response to retinal ischemia is comparable in many ways to that of ischemic stroke. It is therefore reasonable to speculate that stroke-induced retinal injury shares similar inflammatory pathogenesis with cerebral ischemia and retinal ischemia. The NLR pyrin domain containing 3 (NLRP3) inflammasome is an important activator of inflammation which mediates the maturation and secretion of active pro-inflammatory cytokines such as interleukin 1β (IL-1β) and interleukin 18 (IL-18; Zhou et al., 2010a; Deng et al., 2019). Previous studies have shown that NLRP3 inflammasome activation participates in the underlying molecular pathway of ischemic stroke in diabetic mice (Hong et al., 2018) and retinal ischemic/reperfusion injury (Wan et al., 2020). However, it is unclear whether NLRP3 inflammasome is involved in stroke-induced retinal injury in diabetes.

Isoflurane (ISO) has been demonstrated to have anti-inflammatory and neuroprotective properties in ischemic stroke (Zhou et al., 2010b; Zhang et al., 2016; Jiang et al., 2017; Guo et al., 2020). Furthermore, a previous study has suggested that ISO can inhibit the activation of the NLRP3 inflammasome (Yin et al., 2016). To investigate whether ISO offers retinal protection against stroke-induced retinal injury in diabetes, we administered ISO prior to middle cerebral artery occlusion (MCAO) in diabetic mice and studied the role of NLRP3 inflammasome activation in the retina. Our results showed that ISO pretreatment ameliorates stroke-induced retinal injury in diabetes *via* inhibition of retinal NLRP3 inflammasome activation. Our results provide a causative pathway for stroke-induced retinal injury in diabetes and suggest a potential therapeutic target for retinal protection in ischemic stroke in diabetes.

## Materials and Methods

### Animal

Male C57BL/6J mice (4–6 w, 14–18 g) were housed in 12 h light/dark cycle conditions and were supplied adequate food and water before the experiment (*n* = 100). All experimental animals were purchased from the Zhujiang Hospital Animal Experimental Center of Southern Medical University. The study was approved by the Medical Faculty Ethics Committee of Southern Medical University. Firstly, we built the type 2 diabetic (DM) mouse model (*n* = 65) and used non-DM mice as control (*n* = 35).

We excluded mice having fasting glucose less than 10.0 mmol/L (*n* = 16). DM mice were randomly assigned to the sham group, MCAO ISO (−) group, and MCAO ISO (+) group (*n* = 11–21).

Mice which died after surgery were excluded in MCAO ISO (−) group and MCAO ISO (+) group (*n* = 2 in each group). Mice were randomly chosen from sham group, MCAO ISO (−) group, and MCAO ISO (+) group (*n* = 4–6) for retinal gene expression. The eyeballs were collected from sham group, MCAO ISO (−) group, and MCAO ISO (+) group (*n* = 7–13) for tissue section and subsequent staining.

### Type 2 Diabetes Mellitus Mouse Model

The type 2 diabetic mouse model was generated as described previously (Lin et al., 2020). Briefly, we first fed each mouse on a high-fat diet (Guangdong Medical Laboratory Animal Center, Guangzhou, China) for 3 w. A dose of 100 mg/kg streptozotocin (STZ, Sigma, St. Louis, MO, USA) was then injected intraperitoneally. Mice were then fed with a high-fat diet for another 4 w. The blood glucose concentration was measured after fasting for 8 h on day 1, 22, 36, and 50. The criterion for a type 2 diabetes mellitus mouse was fasting glucose >10.0 mmol/L. Control (non-diabetic) mice were housed in the same environment, fed a normal diet, and given an equal-volume intraperitoneal injection of vehicle.

### Ischemic Stroke Mouse Model

The ischemic stroke mouse model was generated by MCAO following the protocol of our previous study (Lin et al., 2020). In brief, mice were anesthetized with 2% isoflurane (RWD Life Science Co., Ltd, Shenzhen, China). After a midline neck incision, a 4–0 nylon monofilament (3 cm) with a blunted tip (Sebiona Technology Co. Ltd., Guangzhou, China) was inserted into the origins of the right middle cerebral artery (MCA) through the right external carotid artery to block the blood flow to the MCA. The suture was left in place for 1 h and reperfusion was established after withdrawing the suture. The mice were sacrificed after maintaining reperfusion for 24 h for the subsequent experiments. Sham-operated mice underwent the same procedure without insertion of the monofilament.

### Isoflurane (ISO) Pretreatment

The ISO pretreatment [ISO (+)] group inhaled 2% isoflurane for 1 h which was delivered by oxygen (O_2_) at a rate of 0.2 L/min 24 h before MCAO procedure. The control group [ISO (−)] received the same procedure without ISO pretreatment. ISO pretreatment and control groups were randomly assigned to ischemic MCAO and sham groups.

### Brain 2,3,5-Triphenyltetrazolium Chloride (TTC) Staining

The whole brain was collected immediately after euthanasia. The brain was sectioned into 1 mm thick slices and stained with 2% TTC (BCCC4696, Sigma, St. Louis, MO, USA) for 15 min and then fixed in 4% paraformaldehyde (PFA).

### Hematoxylin and Eosin (H&E) Staining

The ipsilateral eyeball was isolated and fixed in 4% PFA before being embedded in paraffin. Eyeballs were sectioned in the same coronal sections (5 μm thick). H&E staining was employed to assess retinal ganglion cell layer (GCL) cells and inner retinal measurement. The slices were imaged using a bright-field microscope (DM2500 LED, Leica Microsystems, Germany) and analyzed in ImageJ (version 1.49, National Institutes of Health, Bethesda, MD, USA).

### Terminal Deoxynucleotidyl Transferase-Mediated Nick End Labeling (TUNEL) and Immunofluorescence Staining

Mice were perfused with 4% PFA. Eyeballs were removed and fixed with 4% PFA for 24 h. Frozen sections of 10 μm thickness were embedded in optimal cutting temperature compound (OCT, Tissue-Tek, Sakura Finetek, USA). TUNEL staining was performed using in situ Cell Death Detection Kit, Fluorescein (11684795910, Roche, USA). For immunofluorescence staining, the primary antibodies used were as follows: rabbit anti-NLRP3 (PA579740, 1:1,000, Thermo Fisher, USA), mouse anti-Caspase-1 (sc-56036, 1:100, Santa Cruz, USA), and Rabbit anti-IL-1β (12703, 1:100, Cell Signaling Technology, USA). The secondary antibodies used were goat anti-rabbit (A-11034, 1:1,000, Thermo Fisher, USA) and goat anti-mouse (A-11032, 1:1,000, Thermo Fisher, USA). After that, sections were imaged using a fluorescence microscope (TS100, Nikon, Japan) and analyzed using ImageJ.

### Quantitative RT-PCR (qPCR)

qPCR was performed to measure the expression of NLRP3 inflammasome mRNA in the retina. Primer sequences are as follow: NLRP3, ATT ACC CGC CCG AGA AAG G (forward primer); TCG CAG CAA AGA TCC ACA CAG (reverse primer). Caspase-1, AAT ACA ACC ACT CGT ACA CGTC (forward primer); AGC TCC AAC CCT CGG AGA AA (reverse primer). IL-1β, GAA ATG CCA CCT TTT GAC AGT G (forward primer); TGG ATG CTC TCA TCA GGA CAG (reverse primer). β-actin, GTG CTA TGT TGC TCT AGA CTT CG (forward primer); ATG CCA CAG GAT TCC ATA CC (reverse primer). Using RNAiso Plus kit (TaKaRa, 9109, Japan) to extract the total RNA from the right retina. qPCR was applied by the SYBR Green kit (Takara, RR820A, Japan) using 10 μl cDNA. β-actin was used as the housekeeping gene.

### Statistical Analysis

Data are shown as means ± SD and analyzed using Prism7 software (GraphPad7, San Diego, CA, USA). Statistical significances were determined using Student *t*-test (for two groups) or one-way ANOVA followed by a Tukey Kramer *post hoc* test (for three groups) or repeated measures ANOVA followed by a Bonferroni *post hoc* test (groups with repeated measurements). The difference between groups was considered statistically significant if *P* < 0.05.

## Results

### Ischemic Stroke-Induced Retinal Injury in Diabetic Mice

**Figure 1:**
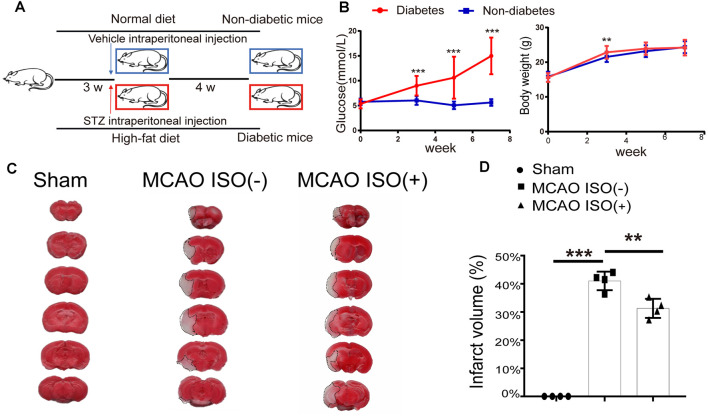
Isoflurane (ISO) pretreatment protects the brain against ischemic damage in a diabetic mouse model. **(A)** Diagram of the protocol used to generate the type 2 diabetic mouse model. **(B)** Continuous monitoring of the blood glucose and body weight changes from the beginning of the high-fat diet (0 week) in diabetic mice and non-diabetic mice. *n* = 24 in each group. **(C)** Representative images of TTC-stained coronal brain slices of sham-operated (left),middle cerebral artery occlusion (MCAO) mice without ISO pretreatment (middle) and MCAO mice with ISO pretreatment (right). The infarct area is indicated *via* the dotted line. **(D)** The infarct volume in the sham-operated, MCAO mice without ISO pretreatment, and MCAO with ISO pretreatment. *n* = 4 in each group. **P < 0. 01, ***P < 0.001.

To assess the retinal injury after ischemic stroke in diabetic mice, we first generated the type-2 diabetic mouse model by administering a high-fat diet and STZ intraperitoneal injection (Figure 1A). Compared to the control group, we found that the blood glucose level significantly increased in diabetic mice at all timepoints (Figure 1B). Although the diabetic mice gained significantly more body weight during the first 3 w, there was no significant difference in body weight compared to the non-diabetic mice after 4 w (Figure 1B).

**Figure 2:**
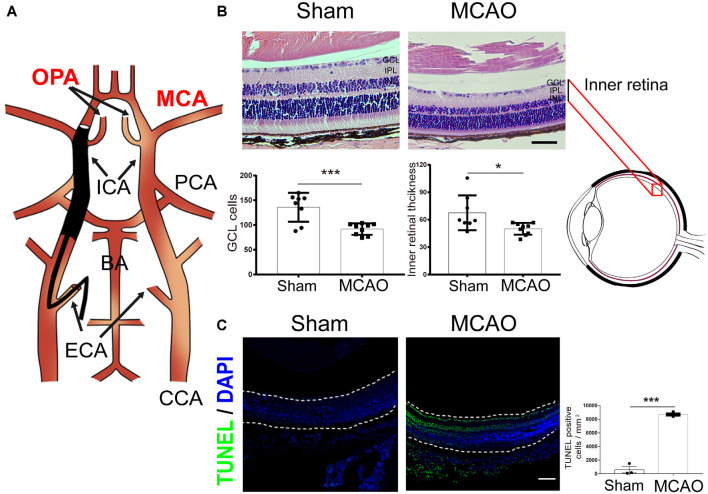
Ischemic stroke induces retinal injury in diabetic mice. **(A)** Schematic diagram of MCAO model blocking the OPA. Monofilament is inserted into the right MCA to block blood flow, thereby blocking the OPA at the same time. **(B)** Top, representative H&E staining in retina post-MCAO. Scale bar, 50 μm. Bottom, bar graphs showing the GCL cell count and the inner retinal thickness in the sham (*n* = 7) and MCAO group (*n* = 9). **(C)** Left, representative images showing TUNEL staining in retina post-MCAO. The retina is indicated between the dotted lines. Green, TUNEL positive cell; Blue, DAPI (magnification, 100×; scale bar, 100 μm). Right, bar graphs showing the TUNEL positive cells density at GCL in the sham (*n* = 3) and MCAO groups (*n* = 4). Sham and MCAO mice were administered with vehicle pretreatment. OPA, ophthalmic artery; MCA, middle cerebral artery; PCA, posterior cerebral artery; ICA, internal carotid artery; BA, basilar artery; ECA, external carotid artery; CCA, common carotid artery; GCL, ganglion cell layer; IPL, inner plexiform layer; INL, inner nuclear layer. *P < 0.05, ***P < 0.001.

In this MCAO model, the ophthalmic artery (OPA) was blocked while the monofilament was inserted into the right MCA (Figure 2A). A previous study has reported a 90% reduction in blood flow in the ipsilateral eye during MCAO (Lee et al., 2020). Correspondingly, when evaluating the histological changes in the retina by H&E staining, we found a significantly decreased ganglion cell layer (GCL) cell count and inner retinal thickness in the MCAO group (Figure 2B). Furthermore, compared to the sham group, MCAO mice exhibited significantly increased apoptotic cells in the GCL as assessed by TUNEL staining (Figure 2C). These results demonstrate that ischemic stroke induces a significant retinal injury in diabetic mice.

### ISO Pretreatment Reduced the Cerebral Damage and Retinal Injury Induced by an Ischemic Stroke in Diabetic Mice

**Figure 3:**
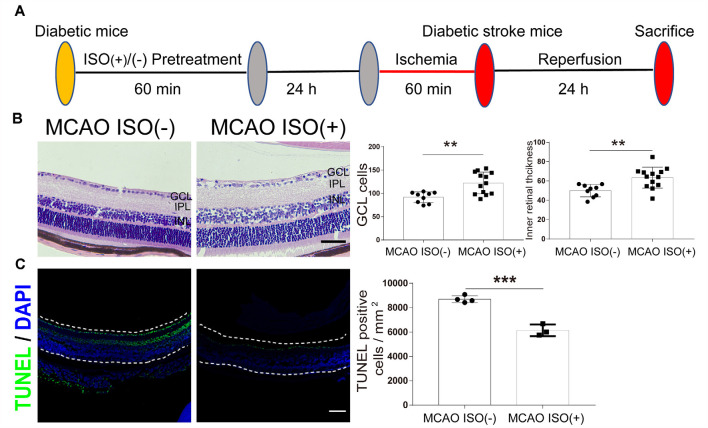
ISO pretreatment reduces retinal injury after ischemic stroke in diabetic mice. **(A)** Diagram of ISO pretreatment protocol. **(B)** Left, representative H&E staining in the retina after MCAO with ISO pretreatment or without ISO pretreatment (magnification, 400×; scale bar, 50 μm). Right, bar graphs showing the GCL cell count and the inner retinal thickness in the MCAO ISO (−) group (*n* = 9) and MCAO ISO (+) groups (*n* = 13). **(C)** Left, representative images showing TUNEL staining in the retina after MCAO with ISO pretreatment or without ISO pretreatment. The retina is indicated between the dotted lines. Green, TUNEL positive cell; Blue, DAPI (magnification, 100×; scale bar, 100 μm). Scale bar, 100 μm. Right, bar graphs showing the TUNEL positive cells density at GCL in the MCAO ISO (−) group (*n* = 4) and MCAO ISO (+) groups (*n* = 3). GCL, ganglion cell layer; IPL, inner plexiform layer; INL, inner nuclear layer. **P < 0.01, ***P < 0.001.

To address whether ISO protects the brain and retinal injury after ischemic stroke in diabetes, 24 h before MCAO onset, we pretreated the diabetic mice with exposure to 2% ISO for 1 h. Mice were then subjected to MCAO, with the MCA occluded for 1 h, and 24 h post-reperfusion, mice were sacrificed (Figure 3A). We confirmed the distinct ischemic brain damage by TTC staining, a redox indicator that distinguishes between metabolically active and inactive tissue. Both diabetic MCAO mice with and without ISO pretreatment exhibited a significant brain ischemic infarct (Figure 1C). However, the infarct volume was significantly decreased in MCAO mice with ISO pretreatment when compared to the MCAO mice without ISO pretreatment (Figure 1D). These data demonstrate the neuroprotective effects of ISO pretreatment in diabetic mice after ischemic stroke.

In addition, the MCAO-induced reduction in GCL cell count and inner retinal thickness were attenuated by ISO pretreatment compared to diabetic MCAO mice pretreated without ISO (Figure 3B). Furthermore, MCAO mice with ISO pretreatment exhibited reduced apoptotic cells in the GCL compared to the MCAO mice without ISO pretreatment (Figure 3C). Our data thereby indicates that ISO pretreatment protects the retina against ischemic injury in diabetic stroke mice.

### ISO Pretreatment Inhibited NLRP3 Inflammasome Activation in Stroke-Induced Retinal Injury

**Figure 4:**
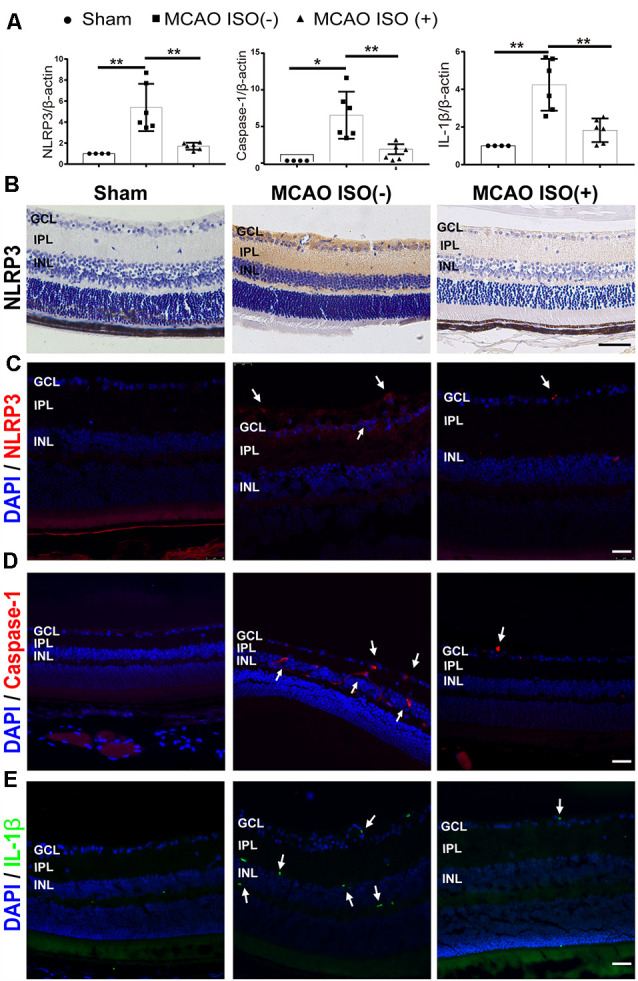
ISO inhibits NLRP3 inflammasome activation in the retina after ischemic stroke in diabetic mice. **(A)** Relative gene expression of NLRP3, caspase-1, and IL-1β in the retina. β-Actin served as an endogenous reference gene (*n* = 4 in Sham group; *n* = 6 in MCAO ISO (−) group and MCAO ISO (+) group). **(B)** Representative immunohistochemical staining for the protein expression of NLRP3 in the retina (magnification, 400×; scale bar, 50 μm). **(C–E)** Representative immunofluorescence staining for the protein expression of NLRP3 **(C)**, Caspase-1 **(D)**, and IL-1β **(E)** in the retina. Arrow, NLRP3 (Red), Caspase-1 (Red), and IL-1β (Green) positive cells (magnification, 400×; scale bar, 25 μm). GCL, ganglion cell layer; IPL, inner plexiform layer; INL, inner nuclear layer. *P < 0.05, **P < 0.01.

Evidence suggests that cerebral and retinal ischemia share acute and chronic pathogenic mechanisms including NLRP3 inflammasome activation, which has been shown to be associated with injury severity (Hong et al., 2019; Wan et al., 2020). We therefore speculated that NLRP3 inflammasome activation may be associated with stroke-induced retinal injury in diabetes. First, we examined the mRNA expression of the NLRP3 inflammasome in the retina. As expected, the mRNA expression of NLRP3, caspase-1, and IL-1β showed more robust elevations in the retina of diabetic MCAO mice compared to sham-operated diabetic mice (Figure 4A). Next, we investigated the effects of ISO pretreatment and found reduced retinal NLRP3, caspase-1, and IL-1β mRNA expression in MCAO mice with ISO pretreatment compared to the MCAO group without ISO pretreatment (Figure 4A). NLRP3 inflammasome protein expression also had a similar trend; compared to sham-operated mice, MCAO increased the NLRP3, caspase-1, and IL-1β protein expression in the retina, which was mitigated by ISO pretreatment (Figures 4B–E). Our results indicate that the suppression of NLRP3 inflammasome activation after ISO pretreatment plays an important role in protecting the retina from stroke–induced retinal injury in diabetic mice.

## Discussion

In this study, we demonstrated that focal cerebral ischemia-induced retinal injury in diabetic mice and ISO pretreatment protected against brain damage and stroke-induced retinal injury. We first revealed that cerebral ischemia increased cell loss and induced apoptosis in the GCL in a diabetic mouse model. We next demonstrated that ISO pretreatment protected the brain indicated by reduced infarct volume and retinal injury as evidenced by preserved cell count and reduced apoptosis in the GCL in the MCAO mouse model. Finally, ISO pretreatment inhibited NLRP3 inflammasome activation in the retina after cerebral ischemia which may underlie its protective effects.

Visual impairment is a significant complication in stroke patients that may exacerbate other stroke-related impairments and impede rehabilitative efforts. The occipital lobe is the visual center and accordingly, posterior cerebral artery (PCA) occlusion-induced occipital infarction has been reported as the most common cause of visual impairment after stroke (Rowe et al., 2013). However, visual impairments have been reported in more than two-thirds of ischemic stroke patients with MCA occlusion (Sand et al., 2018). This suggests that ischemic stroke may induce retinal ischemia, thereby damaging the eyes and visual function directly, rather than impairing the visual center. The retinal blood supply is mainly from the central retinal artery, a branch of the ophthalmic artery (OPA), which is the first branch of the internal carotid artery in mice (Singh and Dass, 1960). Both OPA and MCA are the branches of the internal carotid artery. Due to the anatomical relationship between the OPA and MCA, the OPA is also prone to occlusion when MCA occlusion occurs. Approximately 25% of patients with ischemic stroke have complete occlusion of the OPA, leading to retinal ischemia and subsequent retinal damage (Helenius et al., 2012). Similar effects have been observed in rodent MCAO models, with the blood flow of the ipsilateral eye shown to be significantly reduced once the filament is inserted into the MCA (Steele et al., 2008; Nguyen et al., 2019; Lee et al., 2020). Furthermore, decreased blood flow leads to retinal ischemia with the severity of injury proportional to the cerebral ischemic area (Allen et al., 2014). In order to prevent as well as treat visual impairments after stroke, the underlying mechanism of stroke-induced retinal injury needs to be further investigated. A recent study has shown that mitochondrial dysfunction may be an important cause of stroke-induced retinal injury, and stem cell treatment may be a potential therapy (Nguyen et al., 2019). The present study aimed to identify conserved pathogenic mechanisms between stroke, retinal injury and associated risk factors, such as diabetes.

Diabetes is a prevalent disease and confers a greater risk of stroke and retinopathy occurrence. The risk of ischemic stroke with diabetes is 2.3-fold higher in comparison to non-diabetic patients (Liu et al., 2016; Bragg et al., 2017). Furthermore, diabetic retinopathy is a common complication of diabetes and the leading cause of blindness (Gross et al., 2018). In the diabetic population, approximately one-third have signs of diabetic retinopathy, and a third of these patients have more severe vision-threatening retinopathy (Saaddine et al., 2008). Complex pathophysiological mechanisms including increased production of free radicals, advanced glycosylation end-products, pro-inflammatory signaling, and vascular endothelial growth factor, trigger retinal injury in diabetic status. Diabetic retinopathy-induced microvascular lesions can also cause retinal ischemic damage and contribute to visual impairments (Wong et al., 2016). Therefore, not only is the individual more susceptible to stroke, but the retina may also be more susceptible to stroke-induced retinal injury under diabetic status. Accordingly, in the present study, H&E and TUNEL staining revealed a remarkable retinal injury in the diabetic stroke-induced mouse model.

Cerebral blood flow is often temporarily blocked or reduced in surgical operations, such as cardiac and carotid artery extirpation surgeries, thereby inducing a temporary man-made cerebral ischemic event (Apostolakis and Akinosoglou, 2008). In such cases, it is crucial to use appropriate anesthetic drugs to induce clinical anesthesia and prevent the development of cerebral ischemic injury. The common and widely used clinical anesthetic drug, ISO, has been shown to have a dose-dependent protective effect on the incidence and severity of postoperative ischemic stroke (Raub et al., 2021). ISO preconditioning has furthermore been demonstrated to have significant neuroprotective potency in ischemic stroke in experimental studies. ISO preconditioning alleviated neurological deficits, reduced infarction volume, and attenuated apoptosis in rodent MCAO models *via* inhibiting neuroinflammation (Sun et al., 2015b), attenuating ubiquitin-conjugated protein aggregation (Zhang et al., 2010), and increasing lymphoma-2 expression (Li et al., 2008). Our results correspond well with the previous study, ISO pretreatment shows neuroprotective effects and reduced the infarct volume after ischemic stroke in diabetic mice. *In vitro*, ISO preconditioning provided neuroprotection by regulating the toll-like receptor 4 signaling pathway and inhibiting microglia activation in oxygen and glucose deprivation models (Xiang et al., 2014; Sun et al., 2015a, b). In our study, we provide evidence for the first time that ISO pretreatment exerts protection against retinal injury, specifically inhibiting ischemic-stroke induced retinal injury in diabetic mice.

The robust inflammatory response is an important contributor to the progression of cerebral ischemia, retinal ischemia, and diabetic retinopathy (Anrather and Iadecola, 2016; Capitao and Soares, 2016; Mathew et al., 2019; Yang et al., 2020). The NLRP3 inflammasome, a multimeric protein complex and critical component of the inflammatory response, has been linked to the pathogenesis of all three conditions (Hong et al., 2018; Raman and Matsubara, 2020; Yu et al., 2020). Upon recruitment to the inflammasome, caspase-1 is activated, leading to the release of proinflammatory IL-1β and IL-18, and triggering an infiltration of immune cells and intrinsic cell death mechanisms (Yu et al., 2020). The Nod-like receptor (NLR) signals pathway and NLRP3 inflammasome activation have been proven to play a predominant role in ischemic/reperfusion- induced retinal inflammation by RNA microarray profiling (Wan et al., 2020). Of note, previous studies have shown that inhibiting NLRP3 inflammasome activation is an effective means to alleviate the damage associated with ischemic stroke and retinal ischemia (Hong et al., 2018; Gong et al., 2019). Furthermore, ISO has been demonstrated to inhibit the activation of NLRP3 inflammasomes in acute lung injury (Yin et al., 2016). Our data shows that ISO pretreatment can downregulate NLRP3, caspase-1, and IL-1β mRNA and protein levels in the retina after ischemic stroke, suggesting that the protective mechanisms underlying ISO pretreatment may be related to the direct inhibition of retinal NLRP3 inflammasome activation.

In summary, our study provides important evidence attesting to the close associations between diabetes, stroke, and visual impairments, as well as retinal injury and cerebral ischemia. Our results furthermore suggest that ISO pretreatment can reduce ischemic stroke-induced retinal injury under diabetic conditions by inhibiting NLRP3 inflammasome activation. ISO may represent an important and clinically relevant agent to be used in patients who require general anesthesia and are vulnerable to postoperative ischemic stroke.

## Data Availability Statement

The original contributions presented in the study are included in the article, further inquiries can be directed to the corresponding author/s.

## Ethics Statement

The animal study was reviewed and approved by Zhujiang Hospital Animal Experimental Center of Southern Medical University.

## Author Contributions

F-XL and H-FZ contributed to the study plan and design. H-BL, Y-HL, J-YZ, and W-JG conducted the experiments. AO, Z-JY, Z-PF, and H-SS participated in experimental design and manuscript preparation. F-XL and H-FZ critically revised the work. All authors contributed to the article and approved the submitted version.

## Conflict of Interest

The authors declare that the research was conducted in the absence of any commercial or financial relationships that could be construed as a potential conflict of interest.

## Abbreviations

NLRP3: NLR pyrin domain containing 3
MCAO: middle cerebral artery occlusion
ISO: isoflurane
IL-1β: interleukin-1β
IL-18: interleukin-18
GCL: ganglion cell layer
OPA: ophthalmic artery.
